# Examining Inhibitory Affective Processing Within the Rostral Anterior Cingulate Cortex Among Abstinent Cannabis-Using Adolescents and Young Adults

**DOI:** 10.3389/fpsyt.2022.851118

**Published:** 2022-03-28

**Authors:** Ryan M. Sullivan, Kristin E. Maple, Alexander L. Wallace, Alicia M. Thomas, Krista M. Lisdahl

**Affiliations:** Department of Psychology, University of Wisconsin-Milwaukee, Milwaukee, WI, United States

**Keywords:** cannabis, neuroimaging, adolescents, affective processing, inhibitory control

## Abstract

Cannabis use has been associated with deficits in self-regulation, including inhibitory control. Cannabis users have previously exhibited both structural and functional deficits in the rostral anterior cingulate cortex (rACC), a region involved in self-regulation of emotional response and inhibitory control. The present study aimed to examine whether abstinent cannabis users demonstrated abnormal functional activation and connectivity of the bilateral rACC during an emotional inhibitory processing task, and whether gender moderated these relationships. Cannabis-using (*N* = 34) and non-using (*N* = 32) participants ages 16–25 underwent at least 2-weeks of monitored substance use abstinence (excluding tobacco) and fMRI scanning while completing a Go/No-go task using fearful and calm emotional faces as non-targets. Multiple linear regression and ANCOVA were used to determine if cannabis group status was related to rACC activation and context-dependent functional connectivity, and whether gender moderated these relationships. Results showed decreased bilateral rACC activation in cannabis users during fearful response inhibition, although groups did not show any context-dependent connectivity differences between the left or right rACC during calm or fearful inhibition. Gender findings revealed that cannabis-using females compared to males did show aberrant connectivity between the right rACC and right cerebellum. These results are consistent with literature demonstrating aberrant structural and functional rACC findings and suggest that chronic cannabis use may disrupt typical rACC development—even after abstinence—potentially conferring risk for later development of mood disorders. Marginal gender-specific connectivity findings bolster continued findings regarding female vulnerability to effects of cannabis on cognition and affect. Findings should be assessed in longitudinal studies to determine causality and timing effects.

## Introduction

Cannabis use is becoming more common, with 42% of young adults (aged 19–30) using during 2020 in the United States (1). Adolescents and young adults may be particularly vulnerable to the neurocognitive impact of cannabis use due to ongoing neurodevelopment (2, 3). The frontal executive system is one of the last to develop, taking longer to mature than limbic regions involved in emotion (4). Thus, adolescents and young adults may have an increased likelihood of participating in risky activities, such as substance use (2, 4). In turn, the protracted neurodevelopment occurring during adolescence and young adulthood may leave them more susceptible to the neurocognitive effects of substance use (4).

Δ-9-Tetrahydrocannabinol (THC)—the primary psychoactive component of cannabis—binds receptors in the endogenous cannabinoid (eCB) system (5), namely, the cannabinoid receptor-1 (CB1) in the brain. CB1 receptor density is high in the prefrontal cortex (PFC), cingulate cortex, hippocampus, amygdala, basal ganglia, and cerebellum (6–9). The eCB system modulates a variety of functions (e.g., sleep, pain, inflammation, and energy intake), including stress and emotional regulation (10, 11), and—from a psychological perspective—the eCB system is involved in executive functioning, affective processing, and mood disorders (11, 12). Therefore, it is posited long-term cannabis use has the potential to disrupt eCB activity and in turn, impacting these domains.

Inhibitory control, or response inhibition, is conceptualized as withholding a prepotent response (13–16). With few exceptions (17), chronic cannabis exposure has been associated with inhibitory control deficits such that, cannabis users perform more poorly than non-users on behavioral and neuropsychological inhibitory control tasks (18–24); additionally, functional magnetic resonance imaging (fMRI) studies have revealed greater activation in cannabis users versus non-users within inhibitory control regions (25–27). While completing inhibitory control tasks (e.g., Go/No-go or Stop-signal), cannabis users demonstrate hyperactivity of dorsolateral prefrontal, medial frontal, inferior frontal gyrus, cingulate, inferior, and superior parietal, suggesting increased brain processing effort was necessary to achieve inhibition (25–27). Along with task-based fMRI investigations, chronic cannabis use has also been associated with greater functional connectivity during inhibitory control tasks. Cannabis-dependent individuals demonstrated hyperconnectivity between frontal control and substantia nigra/STN network during response inhibition (28) and had greater connectivity between the PFC and occipitoparietal cortex related to task difficulty despite no performance differences (29). Therefore, prior research has suggested that regular cannabis use is linked with increased connectivity across the cognitive control network, including prefrontal regions.

One posited mediator for the impact of cannabis use on inhibitory control is the anterior cingulate cortex (ACC). Cannabis users exhibit ACC hypoactivity associated with reduced error awareness (30) and during response inhibition (i.e., Stroop)—despite similar behavioral performance (31, 32)—along with dispersed ACC activity during behavioral inhibition (33); these findings are evident after minimal to no periods of cannabis abstinence. Specifically, the ACC is critical in automatic emotion regulation (34, 35), which has connections to both the amygdala and the PFC (36–38). Notably, two functionally distinct subdivisions of the ACC exist (36, 37) which each serve unique roles in emotion processing (39). Specifically, the rostral-ventral ACC (rACC) plays a role in regulating emotional conflict, while the caudal-dorsal ACC (dACC) plays a more significant role in emotional appraisal and expression (39, 40). As it relates to cannabis use, acute THC administration demonstrates aberrant limbic, frontal, temporal, ACC, and parietal activity elicited by emotional faces (41–44), and increased connectivity between the rACC/medial PFC and the amygdala (45). Among chronic users, reduced amygdala activation (46–48) and attenuated ACC activation is observed when viewing negative faces and scenes (46, 48). Further, aberrant frontolimbic connectivity (49) and hyperconnectivity between the ACC and parietal, post-central, and precuneus cortices has been demonstrated when responding to cannabis-cues (50). Correspondingly, bilateral rACC hyperconnectivity at rest is associated with increased depressive mood symptoms in cannabis users (51). Moreover, these activation and connectivity findings are demonstrated across varying lengths of sustained abstinence (no abstinence to 7-days) and thus, clarifying the role of longer lengths abstinence in the relationship between ACC and repeated cannabis use is warranted.

Interestingly, cannabis use is associated with structural abnormalities in the ACC among ROI-specific analyses: smaller volumes in individuals with (52, 53) and without (54) psychosis, and reduced cortical thickness in those with concurrent psychopathology (55, 56) and alcohol use (57). Though, whole-brain morphometry findings tend to find null or mixed structural differences in the ACC (58–60). Notably, these varied structural differences in the ACC may be better explained by underlying functional aberrations. Within cannabis users, we previously reported that smaller rACC volumes were linked with deficits in discriminating differences in facial emotions (54), aligning with previous reports (40). Taken together, research indicates that within the ACC, cannabis users generally exhibit reduced gray matter volume and abnormal activation during cognitive control and emotion processing tasks. Additionally, cannabis and other substances have been associated with increased functional connectivity between the ACC and other cortices, though the literature is not consistent, possible indicating increased connectivity in cannabis users is necessary in order to achieve a behavioral response commensurate with controls.

Investigating brain-behavior associations within fMRI investigations can also shed light on applicability of neuroimaging findings on behavior. Cannabis use has been related to increased levels of perceived stress, and on the flip side, lower distress tolerance (61–64). Notably, emotion regulation is a protective factor against the initiation of substance use (65) and can be dysregulated in substance users (66, 67) which, in turn, mediates the relationship between stress and using cannabis as a coping mechanism (68). Therefore, the literature suggests a relationship between cannabis use, increased perception of stress, poor emotional inhibition, blunted rACC activation, aberrant rACC frontolimbic connectivity, and increased rACC connectivity diffusely across cortices.

There is also a question on whether gender may moderate this relationship, as indicated by studies highlighting how females typically outperform males in response inhibition in healthy populations (69–71). Moreover, males and females also differ in basic emotional processing; each gender demonstrates unique neural activation patterns while viewing emotional faces (72–75). Specifically, males exhibit increased frontal and parietal recruitment while females demonstrate greater temporal, ACC, and limbic activation to emotional faces (75–78). Specific to cannabis, preclinical studies indicate eCB activity varies by sex in a regionally specific manner (79) and density is modulated by estrogen activity (79–81). Importantly, females may be more vulnerable to the effects of cannabis on affective circuitry. Female, relative to male, rats are more susceptible to the anxiety and depression producing properties of THC (82) and exhibit greater CB1 desensitization to THC in frontolimbic regions (82, 83). In humans, female cannabis users have volumetric differences in the PFC, amygdala, and ACC relative to non-using females, that are related to subtle but significant behavioral outcomes (54, 84–86) and demonstrate more pronounced differences in left rACC volumes, which is associated with poorer facial emotion processing (54). These findings remain even after variable periods of sustained abstinence (up to one month) and further reinforce investigating potential long-term effects after a period of sustained abstinence. Additionally, given the aforementioned gender differences, there may be neural differences in the left rACC during emotional response inhibition in cannabis using males versus females.

The aim of the present study is to examine rACC activation during inhibitory processing while viewing negative affective stimuli among abstinent adolescent and young adult regular cannabis users and non-users and investigate functional connectivity differences between the rACC and the rest of the brain during this task. We expect to see decreased rACC activation during correct Fearful No-go trials (46, 48) and increased left and right rACC connectivity with frontal and limbic regions (28, 29, 50, 87, 88) among cannabis users, despite abstinence status. In addition, gender differences on these associations will be examined, with females hypothesized to show pronounced differences in left and right rACC activity and connectivity (54, 82–85). Exploratory analyses will examine the correlation between significant findings and perceived stress.

## Materials and Methods

### Participants

All study protocol was approved by university IRB and in accordance with the Declaration of Helsinki. Participants were individuals ages 16–25, recruited from a larger parent study (R01DA030354; PI: Lisdahl). Exclusionary criteria consisted of magnetic resonance imaging (MRI) contraindications, pregnancy, left-handedness, birth complications, traumatic head injury, neurological disorders, learning and intellectual disabilities, vision or hearing impairments, current psychotropic medication use, independent Axis I DSM-IV-TR diagnosis, ≥10 cigarettes per day, and excessive other drug use (>25 lifetime uses of non-cannabis or non-alcohol substance use).

To be included in the cannabis-using group, individuals were required to have used at least 40 times in the past year and have at least 50 lifetime uses. To be included as a non-using control, individuals were required to have fewer than 5 past year and 20 lifetime cannabis uses (86).

### Procedure

Individuals were recruited through flyers posted in the community. Phone screening was conducted to determine eligibility of interested individuals. During screening, demographics, lifetime substance use history was gathered using the Customary Drinking and Drug Use Record (CDDR) (89, 90), and a DSM-IV-TR semi-structured interview, the Mini International Psychiatric Interview (MINI) (91) was administered.

Eligible participants completed an informed consent/assent process. For those under age 18, parent permission and minor assent were obtained. Prior to MRI scanning, participants underwent at least 2-weeks of monitored abstinence of all substances except nicotine (including alcohol and cannabis) using urine toxicology (One Step Drug Screen Test Dip Card Panel; Innovacon, Inc., San Diego, CA, United States) and continuous sweat toxicology (PharmChek Drugs of Abuse Patch; PharmChem Inc., Fort Worth, TX, United States). Participants also underwent repeated breathalyzer testing (Alco-Sensor IV; Intoximeters, Inc., St. Louis, MO, United States) for recent alcohol use. Abstinence was verified at weekly sessions during the 3 weeks preceding the MRI scan.

### Measures

#### Substance Use

Participants were administered the Timeline Follow Back (TLFB) (19, 92), a measure of past year substance use that uses holidays and other memory cues. Substance use was measured in standard units [alcohol (standard drinks), nicotine (number of cigarettes and hits of chew/snuff/pipe/cigar/hookah), cannabis (smoked/vaped flower and concentrates were measured and dosing was converted to joints based grams)] and assessed for each day during the past year. The CDDR was used to measure lifetime and past 3-month substance use (90).

#### Perceived Stress

The Perceived Stress Scale-14 (PSS-14) (93) was used to measure level of perceived stress. The PSS-14 is a 14-item measure of the degree to which individuals perceive situations as stressful and measures levels of distress and ability to cope (94).

### Magnetic Resonance Imaging Data Acquisition

High-resolution anatomical images were collected using a T1-weighted spoiled gradient-recalled at steady-state pulse sequence (TR = 8.2 ms, TE = 3.4 s, TI = 450, and flip angle of 12°). The in-plane resolution of the anatomical images was 256 × 256 with a square field of view (FOV) of 240 mm. One hundred fifty slices were acquired at 1 mm thickness. Echo planar images (EPI) were acquired while performing the emotional Go/No-go task using T2 × weighted gradient-echo EPI pulse sequence (TR/TE = 2500 ms/30 ms, FOV = 200 cm, matrix 64 × 64 voxels, slice thickness = 3.2 mm., flip angle = 90 degrees, 44 contiguous axial slices) with 117 TRs of volume data acquired per run.

### Functional Magnetic Resonance Imaging Task

Participants completed an emotional Go/No-go task previously used with healthy adolescents (95, 96). Task stimuli included fearful, happy, and calm faces from the NimStim set of facial expressions (97). The task included six functional runs, counterbalanced for order, one for each combination of emotion (fearful, happy, calm) and Go/No-go. At the beginning of each run, participants were instructed to press a button (“Go”) for a particular emotional face and to withhold a button press (“No-go”) for a different type of face. On each trial, a face was presented for 500 milliseconds, followed by a jittered intertrial interval ranging from 2 to 14.5 s (mean = 5.2 s). In sum, 48 trials appeared in a pseudorandomized order (35 “Go” and 13 “No-go”).

### Magnetic Resonance Imaging Preprocessing

All images underwent standard preprocessing steps using the Analysis of Functional Neuroimages software package (AFNI) (98). Preprocessing included slice time alignment, motion correction, and co-registration of EPI data to T1 scan with the aid of a cost function. Each voxel’s time series was despiked and a spatial smoothing kernel of 4 mm was used. Each individual’s anatomy was warped to standard (Montreal Neurological Institute; MNI) space and the resulting registration matrix was applied to the EPI data. Then a brain mask was created from the EPI data, which was aligned to the volume with the fewest outliers. Each individual’s activation data was scaled to percent signal change. The first three TRs of volume data were removed from each run. To account for motion, TRs of volume data with greater than 0.4 mm of motion were censored from the analysis. Subjects with >18% of volumes exceeding the 0.4 mm motion threshold on any individual run were removed from the analysis (*N* = 3; cannabis-using male *N* = 1, non-using male *N* = 1, non-using female *N* = 1) (25, 27). Additionally, TRs of volume data with intensity outliers greater than 10% of voxels in the automasked brain were censored. For each fearful and calm No-go stimuli, two functional runs were concatenated together (happy Go/fearful No-go and calm Go/fearful No-go for “fearful No-go”; happy Go/calm No-go and fearful Go/calm No-go for “calm No-go”). Then a gamma function was used for convolution of the stimuli timing to create a Hemodynamic Response Function (HRF). Finally, the HRF was deconvolved with the acquired MRI signal on a voxel-by-voxel basis. Images were visually inspected for accuracy and manually edited when appropriate.

### Region of Interest (Rostral Anterior Cingulate Cortex) Measurement

FreeSurfer’s Desikan-Killiany atlas (99) was used to define the left and right rACC for each participant and was visually inspected for accuracy. The left and right rACC were then used as a seed region for (1) task-based ROI activation analysis and (2) task-based generalized psychophysiological interaction (gPPI) functional connectivity analysis to examine context-dependent connectivity between rACC (left and right) and whole brain.

### Data Analysis

#### Preliminary Analysis

Demographic variables were examined with ANOVA and chi-square analyses. Variables that differed between groups were included in the primary analysis as additional covariates (see Table 1).

**TABLE 1:** Participant demographics, substance use variables, BOLD activation in rACC, and PSS-14 scores.

	Cannabis-using group		Non-using group	
	Females (*n* = 13)	Males (*n* = 21)	Females (*n* = 18)	Males (*n* = 14)
		
	% or *M* ± *SD* (range)	% or *M* ± *SD* (range)	% or *M* ± *SD* (range)	% or *M* ± *SD* (range)
Race (% Caucasian)	46.2%	66.7%	66.7%	78.6%
Ethnicity (% Non-Hispanic)	76.9%	81.0%	77.8%	92.9%
Age (years)	21.4 ± 2.0 (19–25)	21.7 ± 2.0 (18–25)	21.2 ± 2.4 (18–25)	20.9 ± 2.7 (16–25)
Education (years)	14.1 ± 1.3 (12–16)	13.9 ± 1.4 (11–16)	14.2 ± 1.8 (12–18)	14.4 ± 2.4 (9–17)
WRAT-4 Word Reading (raw score)	59.4 ± 6.2 (41–67)	62.6 ± 5.3 (48–69)	61.2 ± 4.1 (53–69)	62.3 ± 4.1 (55–68)
Age of weekly cannabis use onset (years)	17.8 ± 1.3 (16–21)	17.4 ± 1.8 (14–21)	−	−
Lifetime cannabis use (uses)	782.5 ± 625.0 (101–2314)*	1506.5 ± 1666.0 (125–6000)*	1.3 ± 2.5 (0–10)*	1.1 ± 2.0 (0–6)*
Past year cannabis use (joints)	301.5 ± 245.4 (44.7–879.3)*	408.0 ± 529.6 (54.6–2306)*	0.1 ± 0.24 (0–1)*	0.6 ± 1.3 (0–4.8)*
Number of cannabis joints/month in past 3 months	74.9 ± 58.7 (0.2–194.5)*	95.7 ± 107.1 (0–372)*	0.0 ± 0.0 (0–0)*	0.2 ± 0.6 (0–2)*
Length of cannabis abstinence (days)	25.5 ± 6.5 (19–42)	35.3 ± 29.1 (17–150)	−	−
Past year alcohol use (standard drinks)	271.6 ± 290.5 (0–883)*	380.9 ± 312.9 (24–1120.5)	45.3 ± 46.1 (0–137.5)*	179.6 ± 243.5 (0–698.5)
Past year nicotine use	119.4 ± 176.9 (0–626)*	300.9 ± 588.4 (0–1870)	5.5 ± 10.4 (0–30)*	58.3 ± 207.1 (0–777)
Left rACC fearful-calm no-go activation	−0.066 ± 0.12 (−0.3–0.1)	−0.059 ± 0.23 (−0.5–0.3)	0.018 ± 0.14 (−0.2–0.3)	0.088 ± 0.19 (−0.3–0.4)
Right rACC fearful-calm no-go activation	−0.050 ± 0.12 (−0.2–0.1)	−0.063 ± 0.17 (−0.5–0.2)	0.033 ± 0.17 (−0.2–0.6)	0.073 ± 0.25 (−0.5–0.4)
Perceived Stress Scale-14 Total (0–56)	20.9 ± 7.4 (10–34)	16.6 ± 6.3 (6–33)	17.6 ± 4.8 (8–25)	16.7 ± 5.2 (11–30)

WRAT-4, wide range achievement test-fourth edition word reading subtest. *Differences between cannabis users vs. non-user group within gender = p < 0.05.

#### Behavioral Performance

Independent samples *t*-tests were used to compare differences in fMRI task performance between cannabis users and non-users. Participants who achieved <66% accurate “Go” trials for any given run (e.g., happy Go/fearful No-go) were excluded from the analysis due to performance validity concerns (*N* = 4; cannabis-using male *N* = 1, non-using male *N* = 1, non-using female *N* = 2) (100).

#### Primary Analysis

##### Single Subjects Analysis (Activation)

Using AFNI’s 3dROIstats, mean calm No-go activation was subtracted from mean fearful No-go activation for each the left and right rACC, yielding individual fearful-calm No-go activation values.

##### Group Analysis (Activation)

Individual subject fearful-calm No-go activation values for each left and right rACC were entered into SPSS. Multiple regressions were used to determine the relationship between cannabis group status and left and right rACC fearful-calm No-go activation. Main effects and covariates were added into the first block and cannabis × gender interaction was entered into the second block. Gender, past year alcohol use, and past year nicotine use were included as covariates in all analyses.

##### Single Subjects Analysis (Connectivity)

Single subject analysis included linear modeling consistent with gPPI analysis in AFNI. For each subject, four interaction regressors were created (for each combination of left/right rACC and fearful/calm) representing the interaction between changes in the blood oxygen level-dependent (BOLD) response to correct No-go trials and rACC activation. These interaction regressors were each entered into respective linear deconvolution models along with regressors of interest (correct No-go trials, rACC activation) and no interest (motion, drift effect, go trials, inaccurate No-go trials). The deconvolution models were used to determine connectivity within whole brain, yielding regression coefficients for each subject, which were entered into group analysis.

##### Group Analysis (Connectivity)

ANCOVA *via* AFNI’s “3dMVM” was used to perform four group analyses (for each combination of left/right rACC and fearful/calm) on cannabis × gender. Gender, past year alcohol use, and past year nicotine use were included as covariates.

##### Multiple Comparisons

Monte Carlo simulations using AFNI’s 3dClustSim were used to correct for multiple comparisons based on cluster extent (family-wise alpha = 0.05; voxelwise threshold of *p* = 0.005) (101).

#### Secondary Analysis

Pearson correlations were run to investigate whether left or right rACC activation during emotional inhibition was related to perceived stress scale (PSS) total scores. In addition, Pearson correlations were run to investigate whether functional connectivity (regression coefficients) in clusters that significantly differed according to only group and/or group × gender were significantly associated with perceived stress scale (PSS) total scores. Analyses were conducted in SPSS and statistical decisions were made if *p* < 0.05.

## Results

### Preliminary Analysis

A total of 66 individuals (34 cannabis users and 32 non-users) were included in the final analysis. ANOVAs and chi-square tests revealed no significant difference between cannabis users and non-users in age (*p* = 0.37), race (*p* = 0.47), ethnicity (*p* = 0.42), gender (*p* = 0.14), education (*p* = 0.51), reading ability (*p* = 0.81), and PSS-14 total scores (*p* = 0.49). As expected, cannabis users and non-users significantly differed on measures of lifetime cannabis use (*p* < 0.001), past year cannabis use (*p* < 0.001), past year alcohol use (*p* < 0.001), and past year nicotine use (*p* = 0.02). Follow-up demographic analyses examined differences in these variables by gender and by cannabis-group status within gender. Broadly, males and females significantly differed on measures of lifetime cannabis use (*p* = 0.05) and past year alcohol use (*p* = 0.02), such that males reported greater use relative to females. Consistent with full group findings, cannabis-using and non-using females significantly differed in lifetime cannabis use (*p* < 0.001), past year cannabis use (*p* < 0.001), past year alcohol use (*p* < 0.01), and past year nicotine use (*p* = 0.01). In addition, cannabis-using and non-using males significantly differed in lifetime cannabis use (*p* < 0.01) and past year cannabis use (*p* = 0.001) (see Table 1).

### Behavioral Performance

There were no group differences in performance during Go trials paired with fearful No-go (*p* = 0.31), Go trials paired with calm No-go (*p* = 0.40), fearful No-go trials (*p* = 0.21), or calm No-go trials (*p* = 0.07).

### Primary Analysis

#### Rostral Anterior Cingulate Cortex Region of Interest Activation

**FIGURE 1:**
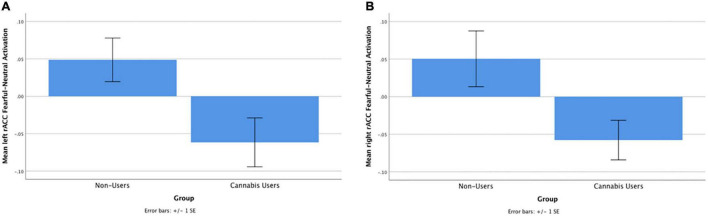
**(A)** Cannabis users, compared with controls, demonstrated significantly less left rACC activation during fearful-calm No-go trials [*t*(48) = –3.08, *beta* = –0.42, *p* = 0.003]. **(B)** Cannabis users, compared with controls, demonstrated significantly less right rACC activation during fearful-calm No-go trials [*t*(48) = –3.07, *beta* = –0.42, *p* = 0.003].

Cannabis users, relative to controls, demonstrated significantly less left [*t*(61) = −3.08, *beta* = −0.42, *p* = 0.003] and right [*t*(61) = −3.07, *beta* = −0.42, *p* = 0.003] rACC activation during fearful-calm No-go (Figure 1). Gender did not moderate the relationship between cannabis group and left [*t*(60) = 0.75, *beta* = 0.09, *p* = 0.46] or right [*t*(60) = 0.55, *beta* = 0.07, *p* = 0.58] rACC activation. Greater past year alcohol use significantly predicted stronger right rACC activation [*t*(61) = 2.13, *beta* = 0.29, *p* = 0.04] (see Table 2).

**TABLE 2:** Regions with significant differences in functional connectivity with the rACC for various contrasts.

Contrast	MNI coordinates x, y, z (mm)	Brain region (s)	Peak *T*-score	Voxel-level significance	Number of voxels	Direction of connectivity
**Left rACC Fearful No-go**						
Alcohol	31.5, 46.5, 73.5	L post-central gyrus	4.19	*p* < 0.001	36	↑alcohol
Right rACC Fearful No-go*						
Left rACC Calm No-go*						
**Right rACC Calm No-go**						
Cannabis: M vs. F	−31.5, 82.5, −40.5	R cerebellum: cerebellar tonsil, pyramis, tuber, inferior semi-lunar nodule, uvula	3.88	*p* < 0.005	89	CAN: M > F

Significant between group differences were determined using a corrected threshold of p < 0.005 determined using a Monte Carlo simulation. Atlas coordinates represent the MNI coordinate system; *no significant clusters.

#### Rostral Anterior Cingulate Cortex Connectivity

##### Left Rostral Anterior Cingulate Cortex Fearful No-Go

Cannabis and cannabis × gender did not significantly predict clusters functionally connected to the left rACC during successful Fearful No-go trials. However, greater past year alcohol use was significantly related to greater left rACC connectivity with one cluster located in the post-central gyrus (Table 2).

##### Left Rostral Anterior Cingulate Cortex Calm No-Go

Cannabis and cannabis × gender did not significantly predict clusters functionally connected to the left rACC during successful Calm No-go trials.

##### Right Rostral Anterior Cingulate Cortex Fearful No-Go

Cannabis and cannabis × gender did not significantly predict clusters functionally connected to the right rACC during successful Fearful No-go trials.

##### Right Rostral Anterior Cingulate Cortex Calm No-Go

**FIGURE 2:**
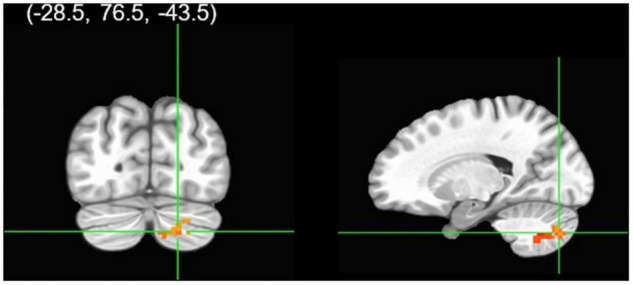
Group analysis of connectivity with the right rACC during correct calm No-go trials. A gender group contrast revealed the right rACC had greater functional connectivity with a cluster in the right cerebellum in males relative to females. The colors represent areas of significant connectivity; warm colors indicate increased connectivity (voxelwise threshold *p* < 0.005; family-wise correction *p* < 0.05).

**FIGURE 3:**
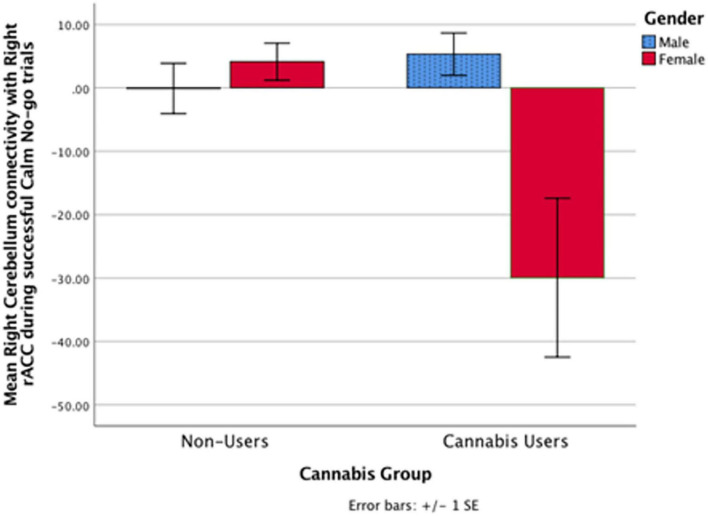
In the cannabis-using group, the right rACC had greater functional connectivity with a cluster in the right cerebellum in males relative to females during successful calm No-go trials (*p* < 0.005). Non-users did not exhibit a significant gender relationship. Notably, there was no significant cannabis × gender interaction.

Cannabis and cannabis × gender did not significantly predict clusters functionally connected to the right rACC during successful Calm No-go trials. Despite no cannabis × gender interaction, within a contrast of cannabis-using males compared to cannabis-using females, males demonstrated significantly greater connectivity relative to females between the right rACC and a cluster in the right cerebellum (Table 2 and Figures 2, 3). Within non-users, gender was not related to right rACC connectivity.

### Secondary Analysis

#### Rostral Anterior Cingulate Cortex Activation and Perceived Stress

Within cannabis users, neither left [*r* = −0.001, *p* = 0.996] nor right [*r* = −0.006, *p* = 0.97] rACC was associated with Perceived Stress Scale total scores.

#### Rostral Anterior Cingulate Cortex Connectivity and Perceived Stress

Right cerebellum and right rACC connectivity were not significantly related to perceived stress within the cannabis-using group [*r* = −0.20, *p* = 0.25], in neither males [*r* = −0.17, *p* = 0.45] nor females [*r* = −0.01, *p* = 0.97] for rACC calm No-go connectivity finding.

## Discussion

The current study is the first to assess relationships between cannabis use, gender, and rACC activation and functional connectivity during an emotional response inhibition task. It was found that abstinent cannabis users, relative to non-using controls, had significantly decreased left and right rACC activation during successful response inhibition upon presentation of fearful faces. Regarding connectivity results, there were no significant differences between abstinent cannabis users and controls in rACC connectivity during fearful or calm response inhibition. Further, group level moderation by gender was non-significant. However, follow-up contrasts revealed that abstinent cannabis-using males, relative to cannabis-using females, demonstrated greater right rACC and right cerebellum connectivity during successful calm No-go trials. Within non-users, gender was not related to rACC connectivity. Interestingly, findings were unrelated to current levels of perceived stress.

The finding that abstinent cannabis users had decreased activation in the right and left rACC during emotional response inhibition complements the ACC structural literature, which has shown relationships between cannabis use and reduced volume and thickness in the ACC (52–57, 102). In conjunction, previous functional studies have also found relationships between cannabis use and reduced ACC activation in response to emotional content and stress (46, 48, 103). Contrarily, Wetherill et al. (104) showed that cannabis users have greater perigenual ACC activation in response to backward masked aversive stimuli. Notably, participants were slightly older, non-abstinent (mean = 1.5 days abstinence), treatment-seeking cannabis users (104), which differs from the abstinent adolescent-young adult users in the current study. Studies have also demonstrated ACC hypoactivation or different distribution of ACC activation in cannabis users during response inhibition tasks without an emotional component (30–33). The present study builds upon these two bodies of literature by showing that cannabis users, following a monitored at least 2-week abstinence period, had rACC hypoactivity during fearful response inhibition after controlling for calm response inhibition.

Similarly, Dreyfuss et al. (78) found that in healthy individuals, right ACC recruitment was associated with successful inhibition of response to fearful non-targets; consistent with the present study’s finding. The current findings extend this literature by demonstrating that controls—compared to cannabis users—recruit the rACC more during fearful relative to calm response inhibition. This suggests that cannabis users do not recruit the rACC to the same extent as controls when successfully inhibiting a motor response when processing a fearful face. Thus, cannabis users may rely on other networks during emotional response inhibition that are hyperactive relative to controls during response inhibition or emotion tasks (25–27, 49, 105), which may serve as a consequence of or a risk for problematic use (106, 107). It is possible that rather than recruiting the rACC during emotional response inhibition, cannabis users recruit areas (e.g., right dorsolateral prefrontal, medial frontal, superior and inferior parietal, right occipital, posterior cingulate, precuneus) that are hyperactive during neutral response inhibition and emotional processing paradigms. A whole-brain activation study would be necessary to address which regions cannabis users recruit during emotional response inhibition. Further, longitudinal studies on ACC development in conjunction with cannabis use are needed to disentangle directionality.

During adolescence and young adulthood, the ACC undergoes significant structural and functional development (108–111). Given the current study’s findings, in conjunction with previous literature showing ACC structural and functional differences, chronic cannabis use may interfere with normal ACC development. The proposed mechanism behind this is a reduction in CB1 receptors within the cingulate cortex associated with chronic cannabis use (112), positing the ACC as particularly vulnerable due to its high density of CB1 receptors (6, 7). Moreover, endocannabinoid signaling *via* CB1 receptors in the mPFC (including the rACC) regulates the HPA stress response (10, 113). Therefore, damage to the endocannabinoid system *via* long-term cannabis use may result in abnormal or decreased neural response to aversive stimuli. This is consistent with research suggesting that abnormal adolescent development of ACC structure and function is associated with depression and anxiety (111, 114–116). In the current study, contrary to prediction, decreased rACC activation in cannabis users was not associated with perceived stress and coping ability. It is possible that the neural differences observed in cannabis users during functional MRI precede differences in perceived stress that have yet to emerge, particularly given the study’s exclusion for mood disorders. A longitudinal study would be necessary to address whether decreased rACC activation in abstinent cannabis users predicts emergence into mood disorders.

While decreased rACC activation was observed in abstinent cannabis users, no relationship was then found between rACC connectivity with other cortical regions by cannabis-group status for either fearful or calm response inhibition. Based on previous findings of greater functional connectivity in cannabis users during response inhibition tasks (28, 29, 50, 87, 88), it was hypothesized that greater connectivity in cannabis users would be necessary to achieve successful response inhibition. Perhaps cannabis users had decreased activation in other areas in addition to the rACC, but to different extents, such that no increase or decrease in rACC connectivity relative to controls was apparent. Additionally, in abstinent cannabis users, other regions might compensate for aberrant rACC activation in a manner that results in similar connectivity and performance across both cannabis users and controls. A whole brain activation study would provide insight into the behavior of other regions in abstinent cannabis users during emotional response inhibition. Regions with significant activation in a whole brain analysis could be more appropriate than the rACC as seeds for an ROI connectivity analysis.

While gender did not significantly moderate the relationship between cannabis use and rACC connectivity during either fearful or calm response inhibition, within a contrast of only the cannabis-using group, gender was related to rACC connectivity. Specifically, cannabis-using males, relative to females, demonstrated greater connectivity between the right rACC and right cerebellum during Calm No-go response inhibition. Because no gender interaction was observed, this is considered a marginal finding useful for future directions and should be replicated. On inspection (Figure 3), males’ right rACC and right cerebellum connectivity during Calm response inhibition more closely resembles connectivity of male and female controls. This marginal finding suggests that females may be more vulnerable to the effects of cannabis on cognition and affect, consistent with previous research (54, 82, 84–86). This may potentially be due to differences in gender-specific use patterns (117), though, warrants future investigation in larger sample sizes that can conduct gender-specific analyses. Related to the present findings, the cerebellum has a particularly high density of CB1 receptors and female rats have exhibited increased vulnerability to reduced CB1 receptor expression in the cerebellum under repeated stress (6, 7, 118). Alternatively, it is possible that increased rACC and cerebellum connectivity in cannabis-using males indicates an over-reliance on the cerebellum, which has been observed in substance users during other cognitive tasks (119–122). Thus, cannabis use may differentially impact male and female rACC-cerebellar circuitry during response inhibition. Again, this finding should be replicated in a larger sample.

There are important limitations that possibly contribute to the null connectivity findings. Length of abstinence was relatively long in the current study compared with most studies in the extant literature [mean = 31 days vs. 3 days in most studies of emotion and neurocognition in cannabis users, e.g., Gruber et al. (46); Wesley et al. (48); Zimmermann et al. (49)], thus previously reported findings may reflect fairly acute effects that recover with sustained abstinence. Then, a more lenient decision on individual voxel thresholding (set at *p* < 0.005) was made to detect more subtle effects (e.g., small to medium effect sizes), yet, research has shown that thresholding at *p* < 0.001 decreases the likelihood of false positives and bolsters fMRI finding interpretation (123). Thus, findings will need to be replicated in a larger sample in order to detect small effect sizes. Notably, the current study demonstrated decreased rACC activation during emotional response inhibition, even after controlling for calm response inhibition. Thus, the activation results in the current study are consistent with each the emotion processing and response inhibition literatures (30–32), and in turn, may generalize to passively viewing fearful faces or other negatively valenced stimuli (46, 48, 104). Further, as response inhibition impairments may be a risk factor for problematic substance use (106, 107, 124), these findings bolster this literature, yet, the current study is cross-sectional and cannot determine causality of the relationship between cannabis use and inhibitory control. Therefore, longitudinal research is warranted on the causal relationship between cannabis use and inhibitory control, as well as the underlying neural mechanisms.

In the current study, cannabis users exhibited decreased right and left rACC recruitment during successful response inhibition to fearful faces compared to calm faces. These findings build upon previous studies demonstrating ACC hypoactivation during emotion processing and inhibitory control tasks (30–32, 46, 48, 104). Chronic cannabis use during adolescence may interfere with typical development of the rACC and other brain regions important for emotion regulation or may pose as a risk-factor for subsequent use; prospective, longitudinal studies are best-suited to disentangle this relationship. Abnormal ACC development is related to depression and anxiety (111, 114–116). Therefore, rACC functional differences may subsequently emerge into mood differences, predisposing cannabis users to later development of affective disorders. Given the significant development of the rACC during adolescence and potential impact of chronic cannabis use, this region may be important to track structurally and functionally over time in prospective longitudinal studies.

## Data Availability Statement

The raw data supporting the conclusions of this article will be made available by the authors, without undue reservation.

## Ethics Statement

The studies involving human participants were reviewed and approved by University of Wisconsin-Milwaukee Institutional Review Board. Written informed consent to participate in this study was provided by the participants’ legal guardian/next of kin.

## Author Contributions

RS: conceptualization, formal analysis, data curation, writing-original draft, and writing-review and editing. KM: conceptualization, investigation, and writing-review and editing. AW: software, data curation, and writing-review and editing. AT: methodology, software, data curation, and investigation. KL: conceptualization, methodology, investigation, resources, writing-review and editing, project administration, and funding acquisition. All authors contributed to the article and approved the submitted version.

## Conflict of Interest

The authors declare that the research was conducted in the absence of any commercial or financial relationships that could be construed as a potential conflict of interest.

## Publisher’s Note

All claims expressed in this article are solely those of the authors and do not necessarily represent those of their affiliated organizations, or those of the publisher, the editors and the reviewers. Any product that may be evaluated in this article, or claim that may be made by its manufacturer, is not guaranteed or endorsed by the publisher.
